# Responses of Root Endophytes to Phosphorus Availability in Peach Rootstocks With Contrasting Phosphorus-Use Efficiencies

**DOI:** 10.3389/fpls.2021.719436

**Published:** 2021-09-27

**Authors:** Yu Zhang, Xin Liu, Jiying Guo, Jianbo Zhao, Shangde Wang, Zhiqin Zheng, Quan Jiang, Fei Ren

**Affiliations:** ^1^Beijing Academy of Forestry and Pomology Sciences, Beijing, China; ^2^Key Laboratory of Biology and Genetic Improvement of Horticultural Crops (North China), Ministry of Agriculture and Rural Affairs, Beijing, China; ^3^Beijing Engineering Research Center for Deciduous Fruit Trees, Beijing, China

**Keywords:** peach rootstock, phosphorus deficiency, root endophytes, bacterial community, fungal community

## Abstract

Phosphorus (P) is an important macronutrient for all lives, but it is also a finite resource. Therefore, it is important to understand how to increase the P availability and plant uptake. The endophytes can help host plants to improve P uptake and will be apparently affected by plant genotypes. To investigate the mechanism of root endophytes in promoting P uptake of peach rootstocks, we analyzed the variations of the root endophytic fungal and bacterial communities of peach rootstocks with different P efficiencies under high or low level of P addition. Results showed that Proteobacteria was the dominant bacterial phylum in the roots of all rootstocks under the two levels of P addition. At low P level, the abundance of Actinoplanes in phosphorus-inefficiency root system was apparently higher than that at high P level. Actinoplanes produced important secondary metabolites, improving the stress resistance of plants. Under high P condition, the abundance of Ferrovibrio was higher in Qing Zhou Mi Tao than in Du Shi. Fe oxides considerably reduced the availability of applied P, which partially explained why the P utilization in Qing Zhou Mi Tao is inefficient. Further, Ascomycota was the dominant fungal phylum in the roots of all rootstocks under different levels of P addition. The fungi community of roots varied in different rootstocks at each P level, but was similar for the same rootstock at different P levels, which indicated that genotype had a greater effect than P addition on the fungal community of peach rootstocks.

## Introduction

Peach (*Prunus persica* L. Batsch) is one of the most commercially important species in the Rosaceae family and includes a wide range of cultivars. Peaches are propagated asexually by grafting. Peach rootstocks used in the production of grafted seedlings are selected based on several factors, including ease of propagation, vigor, nutrient translocation to the fruit, and resistance to pests and diseases (Souza et al., 2019). The root system of the rootstock exchanges materials with the soil environment, and the genetic and physiological characteristics of rootstocks vary greatly. Their root systems exhibit different ability of nutrient absorption and translocation, which leads to changes in the growth and development of the scion. Therefore, it is important to study the differences in nutrient uptake and utilization between root systems of different rootstocks to better understand the nutrition and development of peach trees and improve the quality of seedlings and fruit.

Phosphorus (P) is one of the least mobile nutrients in soils (Liu et al., 2016a). Phosphorus is present in limited concentration in soil solution, ranging from 0.1 to 10 micromoles (Hinsinger, 2001). Moreover, due to the heterogeneous distribution of P in soil, some roots still grow in a P-deficient environment even under conditions of sufficient P application. In response to the limited available P in the soil, plants evolved two mainly adaptive mechanisms: One way is to increase the efficiency of phosphorus uptake by roots and the other way is to improve the efficiency of phosphorus utilization in plants (Raghothama and Karthikeyan, 2005; Zhang et al., 2012). Another adaptation is the development of root symbioses that help plants to access otherwise unavailable Pi sources (Crombez et al., 2019). Soil microorganisms play a key role in the transformations of soil nutrients (e.g., nitrogen and P; Hu et al., 2018), and some can form mutually beneficial symbioses with plant roots. These microorganisms play an important role in the supply of soil nutrients to host plants (Jacoby et al., 2017). In grape (*Vitis vinifera*) and tomato (*Lycopersicon esculentum*), rootstock genotype can influence bacterial diversity and composition (Marasco et al., 2018; Poudel et al., 2019). The microbial community structure of apple rootstocks with different P efficiency was also significantly different (Chai et al., 2019).

Although several studies have catalogued the rhizosphere bacterial microbiome or fungal community, which mainly focused on crops. There has been few research on woody plants. The internal fungal and bacterial communities of woody plants remain poorly described. Therefore, the purposes of the present study were (i) to identify the most P-efficient genotype from eight peach rootstocks and (ii) to characterize the fungal and bacterial communities of peach rootstocks with different P efficiencies grown at different levels of P supply. Our findings suggest that the peach root system contains diverse niches that changed dynamically in response to soil nutrient level. It is important to explore the mechanism of efficient soil P utilization by peach rootstocks in order to select and breed highly P-efficient peach rootstock germplasm.

## Materials and Methods

### Plant Materials and Growth Conditions

The experiment was conducted in a greenhouse of Beijing Academy of Forestry and Pomology Sciences, Haidian District, Beijing, China, using eight peach rootstocks: Du Shi, Da Lian Ri Ben, Bei Da Yu, Qing Zhou Mi Tao, Tsukuba 3, Tsukuba 6, San Kuai Shi, and GF677. All plant materials were offered by the National Germplasm Repository for Peach and Strawberry in Beijing. Maturing peaches with uniform size were collected, and the shell was removed to obtain the seeds. The seeds were surface sterilized with 75% alcohol, rinsed three times with distilled water, placed in a petri dish with cotton, and stored at 4°C. After 90days, the germinated seeds were transferred to individual pots. Initially, the plants were irrigated with deionized water every 2days. After 1week, they were irrigated with 200ml of nutrient solution every 2days. The nutrient solution contained either 1mol/L P (high P, HP) or 0mol/L P (low P, LP). The concentrations of the other nutrients were those of standard Hoagland’s nutrient solution. Each treatment was replicated four times. Each treatment includes 20 plants.

### Plant Harvest and Measurements

After 30days, four plants from each of the eight peach rootstocks were selected and divided into shoots and roots. Four plants were selected from each treatment for measurement of plant height. After measuring the fresh weight, all plant samples were placed in a 105°C oven for 30min to deactivation of enzymes and then dried to a constant weight at 75°C. The dried materials were ground to a fine powder through 1mm sieve. Total P concentration of shoots and roots was measured using a modified Kjeldahl digestion and the vanado-molybdate automated colorimetric method described in Peng et al. (2012). For fungal and bacterial community analyses, root samples from six plants were obtained from Du Shi (D) and Qing Zhou Mi Tao (Q) grown at both P levels. Plants were removed from the pots and rinsed with pure water. Fresh root samples were wiped with paper, wrapped it in tin foil, and put immediately into liquid nitrogen. These samples were stored at −80°C for downstream microbiome analysis. According to the growth status and phosphorus utilization efficiency of the eight rootstocks, Du Shi was a phosphorus efficient peach rootstock and the other seven peach rootstocks were phosphorus-inefficient rootstocks. Therefore, in this article, only one phosphorus-inefficient rootstock (Qing Zhou Mi Tao) was selected for comparison with Du Shi to determine the root system microbial community structure.

### Analysis of Root Endophyte Community

#### Extraction of Genomic DNA and Amplicon Generation

Total genomic DNA was extracted from samples using the CTAB method. 1% agarose gel was used to determine DNA concentration and purity. Then, the DNA concentration was diluted to 1ng/ μl with sterile water (Du et al., 2020). 16S rRNA/ITS genes of distinct regions (16S V4-V5, ITS1) were amplified using specific primers with barcodes (Schoch et al., 2012; Chai et al., 2019).

#### Quantification and Purification of PCR Products, Library Preparation, and Sequencing

An equal volume of 1× loading buffer (containing SYBR green) was mixed with the PCR products, and they were separated by electrophoresis on a 2% agarose gel. The PCR products were mixed in equidensity ratios, and the mixed products were purified with a Qiagen Gel Extraction Kit (Qiagen, Germany). The library was sequenced on the Illumina NovaSeq platform to generate 250-bp paired-end reads.

### Statistical and Bioinformatic Analyses

Two-way ANOVA was conducted to test the effects of genotype, P levels, and their interactions on plant physiological traits (plant height, shoot fresh weight, root weight, and P utilization efficiency). The effects of genotype on plant physiological traits were analyzed by using one-way ANOVA, followed by *post-hoc* tests (Tukey’ HSD) in each P addition treatment, and the effect of P addition by t-test in each peach genotype rootstock, respectively.

For microbial community analysis, the raw forward and reverse reads from each sample were joined at their overlapping ends using a minimum overlap of 50 nucleotides and a maximum of one mismatch within the overlapping region with the fastq-join algorithm.^1^ The reads were then quality screened using a minimum PHRED score of Q20. Use UPARSE^2^ to organize the sequence (Caporaso et al., 2010), and the QIIME software (ver. 1.8.0, http://qiime.org) was used to analyze the resulting data. The sequences were clustered into operational taxonomic units (OTUs) at a similarity level of 97% to calculate the Chao1 richness estimator and the Shannon diversity index. The Ribosomal Database Project Classifier tool was used to classify all sequences into different taxonomic groups^3^ (Quast et al., 2013).

Student’s t-tests were performed to compare the bacterial diversity of roots from given rootstocks and P conditions, and MetaStat software (MetaStat, Inc., Boston, MA) was used to compare the fungal diversity. The relative abundances of bacterial and fungal taxa were determined, and statistical analyses were based on the false discovery rate-corrected Kruskal-Wallis test (*p*<0.05). The variations in bacterial and fungal community composition of root were analyzed using the permutational multivariate (PERMANOVA, 999 permutations) and visualized by principal coordinates analysis plots (PCoA, based on the Bray-Curtis distance matrices). All statistical analyses and visualizations were performed in R.4.0.5 (R Development Core Team).^4^

## Results

### Plant Morphological and P Uptake

**Figure 1:**
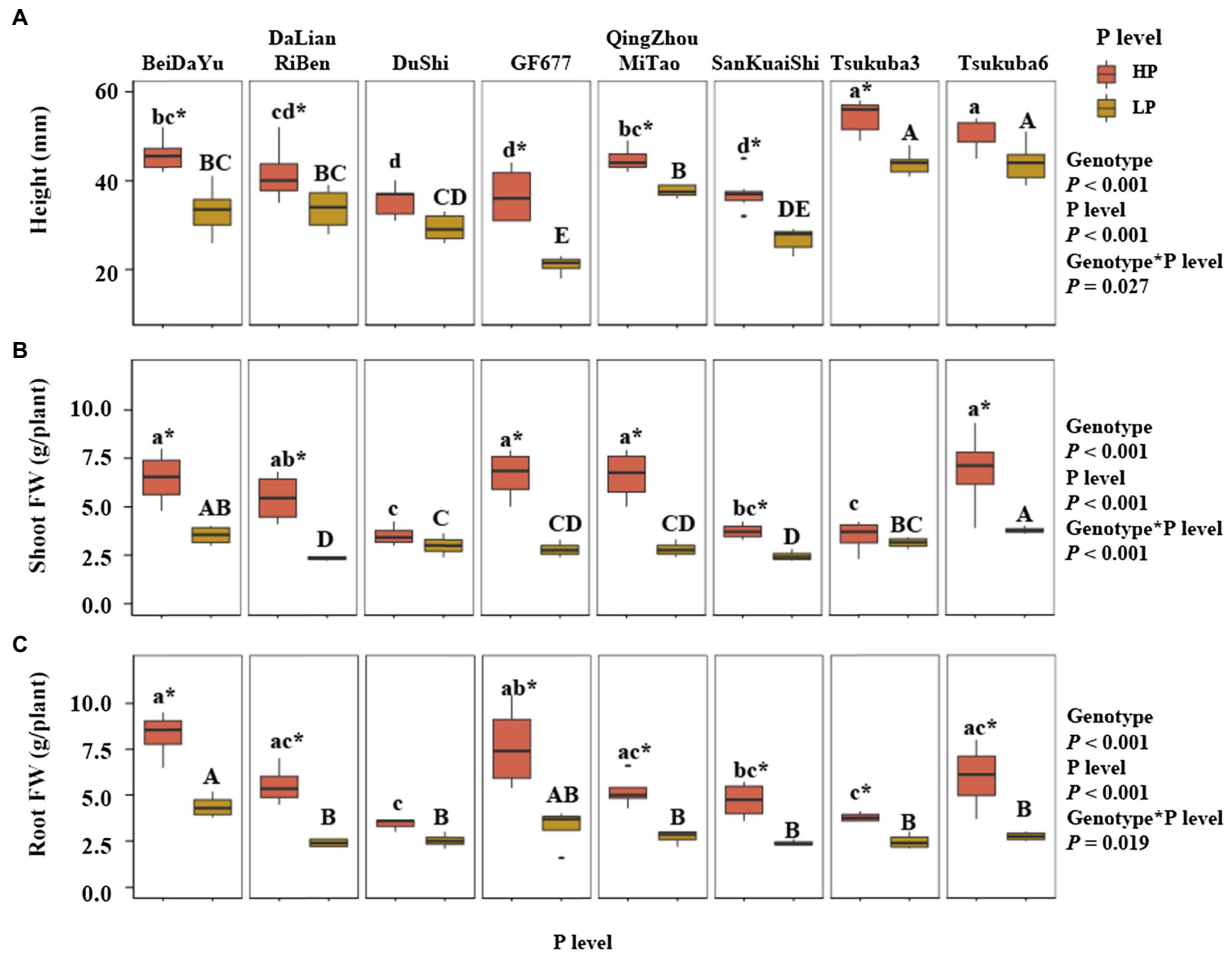
Plant physiological traits at two levels of P supply. **(A)** Height, **(B)** Shoot FW (fresh weight), and **(C)** Root FW of eight peach rootstocks grown under HP or LP conditions. The statistical results are of two-way ANOVA testing for the effects of genotype and P level on the height, shoot FW, and root FW. Different lower- and upper-case letters indicate significantly differences (*p*<0.05) in the growth of eight peach rootstocks under HP and LP, respectively. ^*^ indicates significantly effects of the same peach rootstock species under two levels of phosphorus supply at *p*<0.05 level.

Genotype and P level had an highly significant effect on the height of eight peach rootstocks, and there was a significant effect between genotype and P level on the height (*p<* 0.05; Figure 1A). Under the HP level, the height of Tsukuba 3 and Tsukuba 6 was higher than the other six peach rootstocks, and the heights of Du Shi, GF677, and San Kuai Shi were significantly lower than other peach rootstocks. Under LP level, the height of Tsukuba 3 and Tsukuba 6 was similar to that of HP supply, both of which were significantly higher than the other peach rootstocks. GF677 had the lowest height under low phosphorus conditions. The height of Du Shi was not significantly different at both P levels, and the same was true for Tsukuba 6 (Figure 1A). Significant interaction of genotype and P level was observed for shoot fresh weight (FW; *p*<0.001; Figure 1B). Under HP level, shoot fresh weight of Bei Da Yu, GF677, Qing Zhou Mi Tao, and Tsukuba 6 was significantly greater than that of Du Shi, San Kuai Shi, and Tsukuba 3, but not significantly different from those of Da Lian Ri Ben (Figure 1B). The shoot fresh weight of Tsukuba 6 remained the heaviest under low P treatment. There were no significant differences in shoot fresh weight between both phosphorus supply for Du Shi and for Tsukuba 3 (Figure 1B). Varieties significantly differed in the root fresh weight (Figure 1C). Across all P treatments, the root fresh weight of Bei Da Yu was significantly greater than that of the other seven peach rootstocks (Figure 1C). The interaction of genotype and P level had a significant effect on root fresh weight (*p*<0.05). The fresh weight of roots of Du Shi was not significantly different under the two phosphorus levels, and the root growth of the other seven peach rootstocks was significantly inhibited by low phosphorus availability.

**Figure 2:**
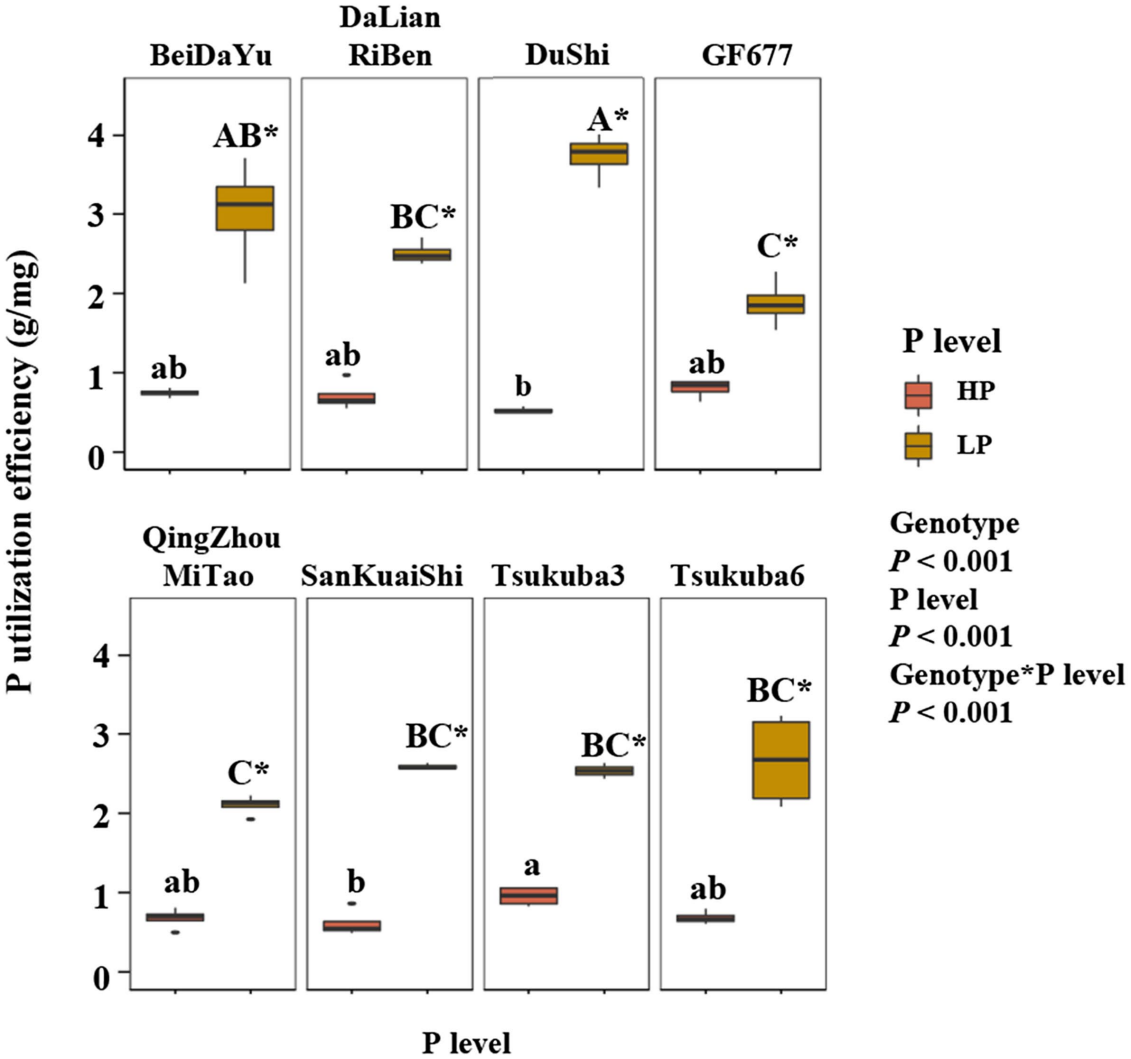
Phosphorus (P) utilization efficiency (PUE) of eight peach rootstocks under HP and LP. The statistical results are of two-way ANOVA testing for the effects of genotype and P level on PUE. Different lower- and upper-case letters indicate significantly differences (*p*<0.05) in PUE of eight peach rootstocks under HP and LP, respectively. ^*^ indicates significantly effects of the same peach rootstock species under two levels of phosphorus supply at *p*<0.05 level.

The level of phosphorus supply had an highly significant effect on P utilization efficiency (PUE) of eight peach rootstock (Figure 2). Phosphorus utilization efficiency was higher in all eight rootstocks under LP conditions than under HP conditions (Figure 2). Genotype effect on PUE was also highly significant, and there were differences in PUE among peach rootstocks of different genotypes at the same level of P supply. The P utilization efficiency of Du Shi was significantly greater than that of the other seven rootstocks under LP. Although PUE of Du Shi was the lowest under high phosphorus conditions, it was the highest when phosphorus deficiency stress was encountered. Across plant growth conditions, the growth of the other seven peach rootstocks was inhibited to different degrees under low P conditions, but the growth condition of Du Shi was not suppressed. In summary, we concluded that Du Shi was a P-efficient rootstock.

### Root Endophytic Bacterial Community Composition Differs Between Rootstocks Under Different P Conditions

**Figure 3:**
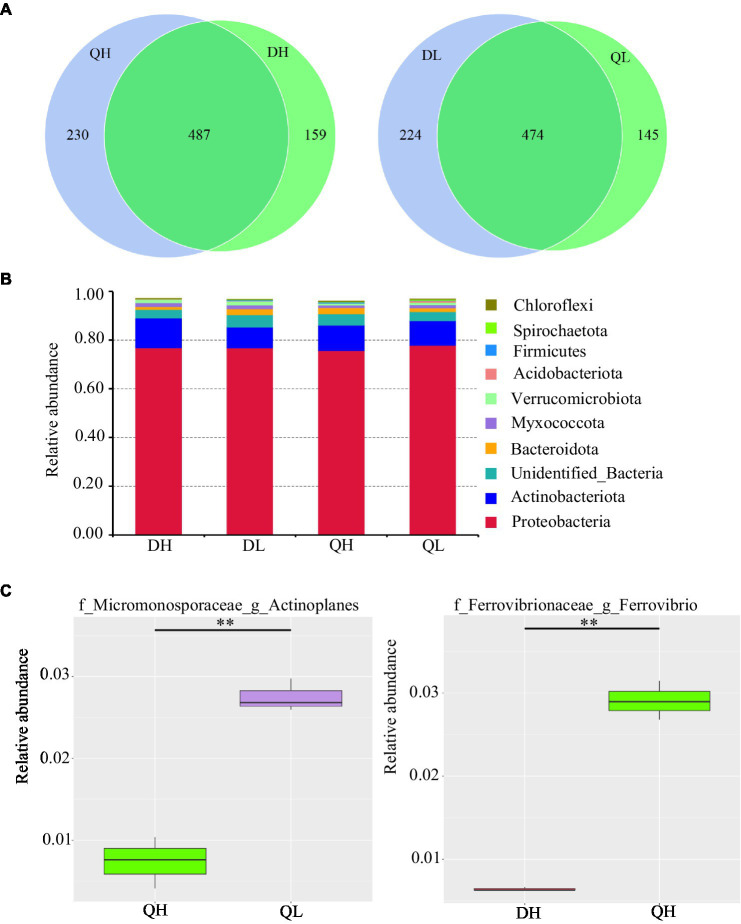
Relative abundance of bacterial taxonomic units in roots of Du Shi and Qing Zhou Mi Tao grown at LP or HP supply. **(A)** Numbers of differentially enriched bacterial operational taxonomic units (OTUs) in Du Shi and Qing Zhou Mi Tao roots. **(B)** Relative abundance of bacteria taxa at the phylum level in Du Shi and Qing Zhou Mi Tao roots. **(C)** Specific genus enriched in Du Shi and Qing Zhou Mi Tao roots under HP or LP conditions. ^*^^*^p<0.01, Student’s *t*-test. DH, Du Shi under HP; DL, Du Shi under LP; QH, Qing Zhou Mi Tao under HP; and QL, Qing Zhou Mi Tao under LP.

Using high-throughput sequencing, we analyzed the root endogenic bacterial diversity and composition of the two rootstocks at two P levels. In total across the two rootstocks, we defined that there were 876 OTUs and 843 OTUs under HP and LP conditions, respectively. Under LP conditions, 224 OTUs were detected only in Du Shi, and 145 were detected only in Qing Zhou Mi Tao, indicating that the two rootstocks had distinct bacterial community structures. Under the conditions of high phosphorus, Du Shi had 159 special OTUs, and 230 OTUs were specific to Qing Zhou Mi Tao (Figure 3A). Good’s coverage values were greater than 99%, indicating that the sequencing depth was sufficient. The Chao1 diversity estimator of the bacterial microbiome ranged from 566.52 to 691.86, and the Shannon diversity estimator ranged from 4.30 to 5.19 (Table 1). To investigate how rootstocks and P levels affect the community structure of root-inhabiting bacteria, we classified OTUs at the phylum level (Figure 3B). Some taxa, such as Proteobacteria, were abundant in all rootstocks and P levels (Figure 3B). Proteobacteria was the dominant bacterial phylum in DH (76.9%), DL (76.9%), QH (75.7%), and QL (77.8%). The relative abundance of Actinoplanes was significantly lower in QH than in QL (Figure 3C). Actinoplanes belongs to the phylum Actinobacteriota. Meanwhile, Ferrovibrio belongs to the Proteobacteria phylum, and its abundance in QH was higher than that in DH treatment (Figure 3C).

**Table 1:** Root bacterial abundance and diversity of peach rootstocks under high P (HP) and low P (LP) conditions.

Sample group	Coverage (%)	Chao 1	Shannon
DH	99.50 ± 0.00a	691.86 ± 145.48a	4.76 ± 0.013a
DL	99.60 ± 0.00a	566.52 ± 26.91a	5.19 ± 0.44a
QH	99.53 ± 0.00a	614.61 ± 69.40a	4.92 ± 0.240a
QL	99.46 ± 0.00a	660.64 ± 66.62a	4.30 ± 0.19a

*Within a column, means followed by the same letter are not significantly different (p<0.05)*.

**Figure 4:**
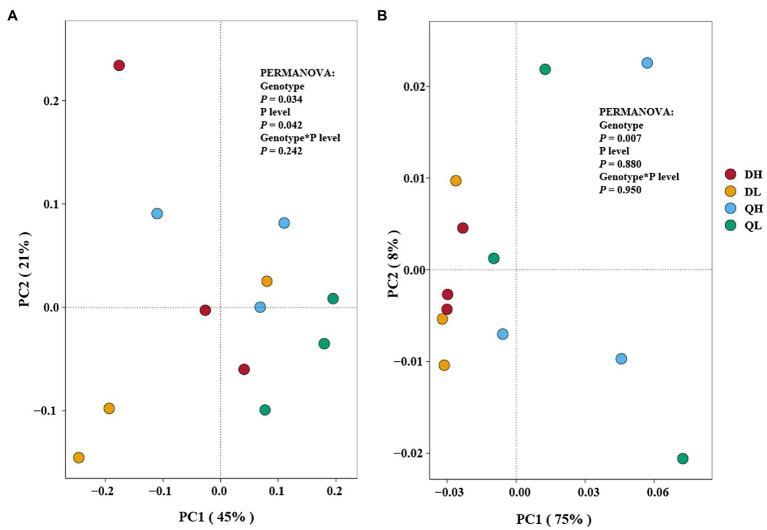
Principal coordinates analysis of Du Shi (D) and Qing Zhou Mi Tao (Q) under HP or LP on the root bacterial **(A)** and fungal **(B)** community composition. The statistical results are of permutational multivariate ANOVA testing for the effects of genotype and P level on bacterial and fungal community composition.

Principal coordinates analysis plots showed that the structure of bacterial and fungal communities differed among DH (Du Shi of high phosphorus), DL (Du Shi of low phosphorus), QH (Qing Zhou Mi Tao of high phosphorus), and QL (Qing Zhou Mi Tao of low phosphorus). The first and second axis accounted for 45 and 21%, respectively, of the total variation in the bacterial communities in the root of Du Shi and Qing Zhou Mi Tao, and the Du Shi was clearly separated from the Qing Zhou Mi Tao (*p*<0.05), which indicated that the community structure of the root bacteria was significantly different between the two rootstocks (Figure 4A). Phosphorus levels had a significant effect on the root bacterial community of peach rootstock (*p*<0.05). The interaction between phosphorus level and genotype had no significant effect on the bacterial community structure in the root systems of the two peach rootstocks (*p*= 0.242).

### Root Endophytic Fungal Community Composition Differs Between Rootstocks Under Different P Conditions

**Figure 5:**
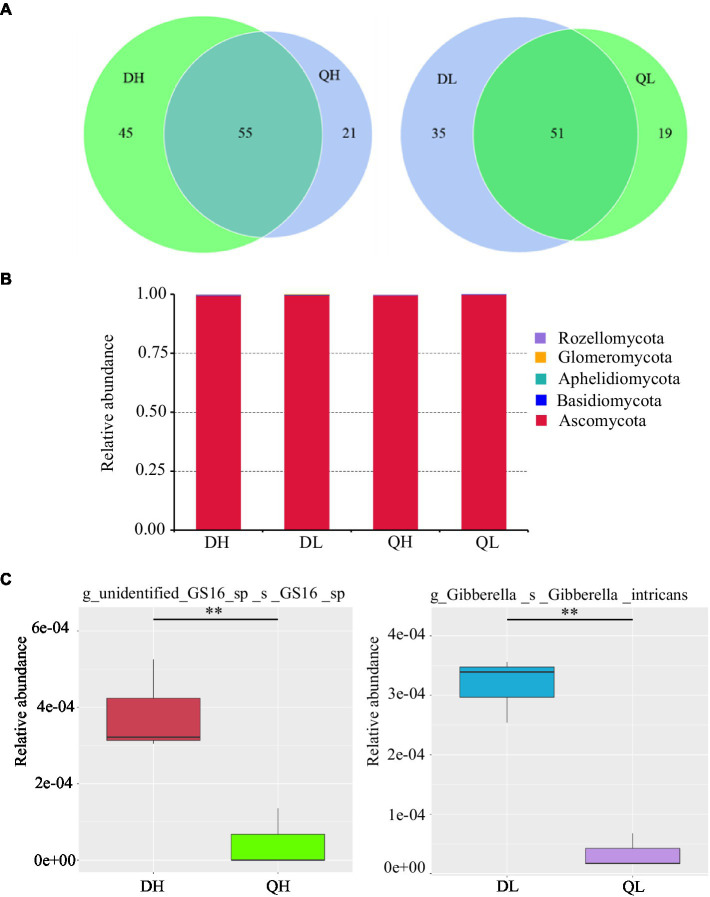
Relative abundance of fungal taxonomic units in Du Shi and Qing Zhou Mi Tao roots grown at high and low P conditions. **(A)** Numbers of fungal OTUs in Du Shi and Qing Zhou Mi Tao roots. **(B)** Relative abundance of fungal taxa at the phylum level in Du Shi and Qing Zhou Mi Tao roots. **(C)** Specific taxa enriched in Du Shi and Qing Zhou Mi Tao roots under HP or LP conditions. ^*^^*^p<0.01, Student’s *t*-test.

There were 45 and 21 unique fungal OTUs in DH and QH roots, respectively, and 35 and 19 unique fungal OTUs in DL and QL roots. DH and QH shared a large number of fungal OTUs (45.5% of total fungal OTUs), and DL and QL shared 48.6% of total fungal OTUs (Figure 5A). Based on the taxonomic affiliations of the OTUs, five fungal phyla were found in the root of peach rootstocks with different P efficiency (Figure 5B). Ascomycota was the dominant fungal phylum in all samples (Figure 5B). GS16, belonged to phylum of Aphelidiomycota, was more abundant in DH roots than in QH roots (Figure 5C). There were no other significant differences in fungal abundance at the phylum level. However, the relative abundance of Gibberella_intricans was significantly greater in DL than in QL at the species level (Figure 5C). Good’s coverage index was greater than 99.9% across all rootstocks and P treatments. Du Shi had a higher value of Chao1 than Qing Zhou Mi Tao under low P conditions, and the Shannon diversity estimator ranged from 0.70 to 0.88 in the fungal microbiome (Table 2).

**Table 2:** Root fungal abundance and diversity in peach rootstocks under HP and LP conditions.

Sample group	Coverage (%)	Chao 1	Shannon
DH	99.99 ± 0.00a	47.13 ± 4.52a	0.78 ± 0.04a
DL	99.98 ± 0.00a	43.12 ± 2.78a	0.70 ± 0.05a
QH	99.99 ± 0.00a	41.88 ± 1.15a	0.88 ± 0.11a
QL	99.99 ± 0.00a	30.27 ± 1.11b	0.83 ± 0.12a

*Within a column, values followed by different letters are significantly different (p<0.05)*.

In addition, PCoA showed that fungal composition of microbial communities differed among all treatments (Figure 4B). It displayed that the first and second axis accounted for 75 and 8%, respectively (Figure 4B). The PERMANOVA results showed a similar result as the bacterial community structure. Genotype had a significant effect on the root fungal community structure of both peach rootstocks (*p*<0.05), while phosphorus levels (*p*= 0.880) and the interaction between the two (*p*= 0.950) had no effect.

## Discussion

### Responses of Different Rootstocks to P Deficiency

Phosphorus is one of the most commonly deficient plant nutrients in soils (Raghothama and Karthikeyan, 2005). Modern agricultural practices are largely reliant on fertilizer applications to ensure high yields, particularly in China orchards (Li et al., 2010). But the situation of phosphate fertilizer is not optimistic. Phosphorus fertilizer is made from the rock phosphate (Cordell et al., 2009). Rock phosphate reserves are expected to be exhausted in the next 50–100years (Johnston et al., 2014). The study suggested that the selected P-efficient genotypes could be used to enhance PUE (Cong et al., 2021). Thus, screening for highly P-efficient genotypes is of great importance (Zhao et al., 2018). One of the characteristics of P-efficient genotypes is high PUE, i.e., the amount of P obtained from the soil that is translocated, remobilized, and utilized for plant physiological processes (Powers et al., 2020). Phosphorus utilization efficiency and relative PUE under low phosphorus condition were used to screen high P efficiency soybean varieties (Wang et al., 2021). In our research, when Du Shi was in sufficient phosphorus supply, PUE was not too obvious. However, PUE of Du Shi increased rapidly when plants grew under low phosphorus stress, making the growth proceed normally (Figures 1, 2). Therefore, Du Shi is a phosphorus efficient peach rootstock that can be directly used for production under low extractable P concentration in soil.

### Root Microbial Community Structure and Taxonomy in Two Peach Rootstocks Under Different P Levels

Previously, much research on root microorganisms of woody plants has focused on the rhizosphere, but few studies have considered the microbes that live inside the root system. For example, the different P efficiency of apple rootstock root system can recruit different kinds of microorganism (Chai et al., 2019). But what about the microbial structure within in the root systems of different rootstocks? In this study, we taxonomically characterized root endophytic microbes in P-efficient and P-inefficient peach rootstocks at two P levels. Bacteria play determining roles in soil and contribute to essential functions in the cycling of nutrient (e.g., nitrogen and phosphorus; Pankratov et al., 2011; Su et al., 2015; Zhang et al., 2019). The results of this study showed that the bacterial community within the root system of peach rootstock was influenced merely by genotype and P levels, and not by the interaction between the two variables (Figure 4A). The bacterial diversity (Shannon index) did not differ significantly between the two rootstocks at each P level (Table 1), but the bacterial community structure differed significantly at the genus level between the rootstocks and P treatments (Figure 3C). The phylum Proteobacteria dominated the root bacterial communities (Figure 3B), consistent with the results of several previous bacterial community studies on crop rhizosphere soils (Mendes et al., 2014; Samaddar et al., 2019), mining soils (Li et al., 2015; Wei et al., 2019), and P-enriched soils (Hu et al., 2020). The Proteobacteria is widespread in the soil environment (Niu et al., 2020). The special Proteobacteria is responsible for converting solar energy into chemical energy through photosynthesis (Berthrong et al., 2014).

At low P level, the abundance of Actinoplanes in Qing Zhou Mi Tao root system was significantly higher than those at high P level. Actinoplanes has two main functions: First, Actinoplanes strains possess a highly sophisticated life cycle that includes differentiating sporangia and motile spores, and second, Actinoplanetes produce a plethora of structurally complex and pharmaceutically important secondary metabolites (Yushchuk et al., 2020). Some secondary metabolites can improve the stress resistance of plants. Under higher P conditions, the abundance of Ferrovibrio was higher in Qing Zhou Mi Tao than in Du Shi (Figure 3C). Ferrovibrio can oxidize Fe(0) to Fe(III) (Zhang et al., 2018; Shi et al., 2019; Liu et al., 2020). Fe oxides in the growing media strongly and negatively affected plant development and P uptake (García-López et al., 2021). Fe oxides considerably reduced the efficiency of applied P (García-López and Delgado, 2016). This is one of the reasons why the phosphorus in Qingzhou Mi Tao is inefficient.

Previous work has documented that there were significantly differences in the fungal communities between root systems (Yu et al., 2017), confirming that roots provide a selective environment for microbes (Bulgarelli et al., 2012; De Souza et al., 2016). Strikingly, Ascomycota accounted for more than 95% of the fungal OTUs across all treatments (Figure 5B). Furthermore, the relative abundance of GS16 (Aphelidiomycota phyla) and Gibberella differed significantly between the roots of Du Shi and Qing Zhou Mi Tao. Aphelidiomycota is not a typical soil and root fungus; little is known about it, and further study is needed (Li et al., 2020). Under low P level, the abundance of gibberellus in roots of Du Shi was significantly higher than that of Qing Zhou Mi Tao (Figure 5C). Gibberellus is a plant rhizosphere growth-promoting microorganism that secretes gibberellin, a plant hormone that promotes plant growth (Yuan et al., 2020). Peach rootstock genotypes influence the endophytic fungal community (Figure 4B). It has been found that significant differences in fungal communities existed in *Arabidopsis* roots and leaves (Bai et al., 2015). The same is true in the stalk of sugar cane (De Souza et al., 2016). In addition, we observed that there were no significant differences in the fungal communities of the same peach rootstock under different P supply levels (Figure 4). In a similar study (Ding et al., 2016), the tall fescue-endophyte-infected varieties in soils had no influence on rhizosphere soil microbial community structure. The structures of AMF communities in maize roots are significantly influenced by growth stage (6-leaf collar, 13-leaf collar, and kernel dough stages), but not by P fertilizer levels (Liu et al., 2016b). Liu et al. (2016b) also indicated that crop phenology may be a stronger determinant than P application in shaping the AMF community structure in roots. One reason why P levels in our study did not affect fungal community may be due to lack of dynamic responses when only sampling at one time point during plant development.

## Data Availability Statement

The original contributions presented in the study are publicly available. This data can be found here: National Center for Biotechnology Information (NCBI) BioProject database under accession number PRJNA739805.

## Author Contributions

YZ conducted the experiments, analyzed the data, and prepared the manuscript. XL analyzed the data. JG, JZ, SW, and ZZ performed the collection and processing of samples and analyzed the data. QJ and RF coordinated the experiments. All authors have read and agreed to the final version of the manuscript.

## Funding

This research was supported by the China Agriculture Research System of Peach (CARS-30), Beijing Academy of Agriculture and Forestry Sciences Innovation Capability Construction Special Project (KJCX20200114), and Beijing Academy of Agriculture and Forestry Sciences Innovation Capability Construction Special Project (KJCX20210701).

## Conflict of Interest

The authors declare that the research was conducted in the absence of any commercial or financial relationships that could be construed as a potential conflict of interest.

## Publisher’s Note

All claims expressed in this article are solely those of the authors and do not necessarily represent those of their affiliated organizations, or those of the publisher, the editors and the reviewers. Any product that may be evaluated in this article, or claim that may be made by its manufacturer, is not guaranteed or endorsed by the publisher.

## Footnotes

^1^ https://expressionanalysis.github.io/ea-utils/

^2^ https://pubmed.ncbi.nlm.nih.gov/23955772/

^3^ http://www.arb-silva.de/

^4^ https://www.R-project.org/
